# Performance of [18F]FDG-PET/CT Imaging in First Recurrence of Invasive Lobular Carcinoma

**DOI:** 10.3390/jcm12082916

**Published:** 2023-04-17

**Authors:** David Bonnin, Sylvain Ladoire, Nathalie Briot, Aurélie Bertaut, Clément Drouet, Alexandre Cochet, Jean-Louis Alberini

**Affiliations:** 1 Department of Nuclear Medicine, Georges Francois Leclerc Research Cancer Center, 21079 Dijon, France; 2 Department of Medical Oncology, Georges Francois Leclerc Research Cancer Center; 21000 Dijon, France; 3 Research Center INSERM LNC-UMR1231, 21000 Dijon, France; 4 Department of Methodology and Biostatistics, Georges-Francois Leclerc Research Cancer Center, 21000 Dijon, France; 5 Laboratoire ICMUB, University Bourgogne, 21000 Dijon, France; 6 Centre Georges-Francois Leclerc, 1 rue du Pr Marion, 21079 Dijon CEDEX, France

**Keywords:** breast cancer, invasive lobular carcinoma, recurrence, PET/CT, 18F-FDG

## Abstract

Background: Invasive lobular carcinoma accounts for 10 to 15% of all breast cancers. The first objective of this retrospective study was to assess the diagnostic performance of FDG-PET/CT scanning in women previously treated for invasive lobular carcinoma with suspected first recurrence. The secondary objectives were to evaluate the impact of PET/CT in a change in treatment and its prognostic value on specific survival. Methods: Patients in whom a PET/CT scan was performed from January 2011 to July 2019 in our Cancer Research Center were enrolled. Recurrence was suspected based on clinical symptoms, abnormal findings on conventional imaging, and/or elevated tumor markers. The diagnosis of recurrence was established by the oncologist after integration of all clinical, biological, histological, imaging, and follow-up data. Prognostic factors of recurrence as predicted by PET were determined using univariate logistic regression. KI67, mitotic index, or grade of mitosis were tested. Survival curves were compared using the log-rank test. Sixty-four patients (mean age: 60.3; SD = 12.4 years) were enrolled. The average time from initial diagnosis of the primary tumor to suspicion of recurrence was 5.2 ± 4.1 years. Forty-eight patients (75%) were judged to have recurrence by the oncologist: 7 local and 41 metastatic, with mainly bone (*n* = 24), lymph node (*n* = 14) and liver (*n* = 10) metastases. Results: Sensitivity, specificity, and positive and negative predictive values of PET/CT to predict recurrence were, respectively: 87%, 87%, 95%, and 70%. SUVmax at recurrence sites was generally high (mean: 6.4; SD = 2.9). False negative PET/CT results occurred with local (*n* = 2), peritoneal (*n* = 2), meningeal (*n* = 1), or bladder (*n* = 1) recurrences. In 40 patients with available histopathological data from suspected sites of recurrence, 30 PET/CT were true positive. In four patients, primary lung (*n* = 1) or gastric (*n* = 1) tumors or lymphomas (*n* = 2) were found. The detection of a recurrence resulted in a change in treatment in 44/48 patients (92%). No association between recurrence predicted by PET and biological biomarkers was found. Median specific survival appears shorter in patients with metastatic recurrence versus patients with local or no recurrence on PET/CT (*p* = 0.067). Conclusions: FDG-PET/CT is an effective and reliable tool for the detection of invasive lobular carcinoma recurrence, although certain recurrence sites specific to this histological type can impair its diagnostic performance.

## 1. Introduction

Breast cancer is one of the most common types of cancer in the world, with more than two million new cases worldwide in 2018 [1]. It is the leading cause of death by cancer in women in Western countries. In more than 95% of cases, malignant lesions of the breast are adenocarcinomas, with two main histological types: ductal and lobular. Invasive lobular carcinoma (ILC) accounts for 10 to 15% of all breast cancers [2], and its frequency has been rising in recent years [3]. The incidence of ILC is highest in women over the age of 50 years, which could explain the increasing proportion of ILC as compared to invasive ductal carcinomas (IDC), in view of the aging of the population [4]. ILC presents several specific challenges, notably the difficulty to detect the primary tumor on imaging, as well as the increased risk of diffuse metastases [5,6], even with histological features that carry a good prognosis (e.g., expression of hormone receptors, low mitotic index). The infiltrating propagation of the tumor cells in a linear fashion (single-file) may explain why ILC is hard to detect by imaging [7], regardless of the type of imaging method used. Positron Emission Tomography/Computed Tomography using 2-deoxy-2-[18F]fluoro-D-glucose (FDG-PET/CT) is sometimes thought to perform less well in the staging of ILC than for IDC, due to the lower FDG-avidity of ILC [8,9,10,11,12,13,14,15]. However, it should be noted that these results were observed in primary tumors and were based on quite small sample sizes since ILC is less frequent. The lower FDG uptake by ILC compared to IDC can be explained by several factors [5]: (i) the tendency of ILC to spread diffusely and elicit little desmoplastic response by the loss of cadherin E expression observed on immunohistochemistry [16]; (ii) the lower tumor cell density, Glut-1 expression, and/or rate of proliferation [10,12,15,17]; and (iii) the higher expression of hormone receptors. It has been shown that on the initial PET/CT scan for staging, the probability of detecting distant metastases was lower for patients with ILC than for those with IDC. In addition, for some ILC patients, distant metastases with poor FDG uptake were only detected on the CT component of the PET/CT scan [18,19].

The main objective of the present study was to assess the diagnostic performance of FDG-PET/CT scanning in women previously treated for ILC and with suspected first recurrence. We also investigated the relationship between PET/CT results, and biological or molecular features of ILC, and whether the detection of a recurrence led to a change in treatment. Finally, the prognostic value for specific survival of detecting metastatic lesions on PET/CT scan was evaluated.

### 1.1. Patients and Methods

In this single-center, retrospective study, we analyzed the medical files of patients followed for ILC at the Cancer Research Center of Dijon, France, from January 2011 to July 2019. Women who had undergone an FDG-PET/CT scan when first recurrence was suspected were eligible for inclusion. Patients with associated IDC, or those who already had local or distant recurrence, were excluded. Histological prognostic factors of the primary tumor, initial treatment, and the reasons leading to a suspicion of recurrence and to the prescription of a PET/CT scan were collected for the included patients.

The final diagnosis of recurrence was established by the oncologist. In charge of each patient, after integration of all clinical, biological, histological, and imaging data within a three-month period from the suspicion of recurrence. Since this analysis was per-patient-based, the confirmation of only one local and/or one metastatic recurrence detected on FDG-PET/CT scan was defined as recurrence with a potential impact on patient management.

### 1.2. Characteristics of Patients and ILC

**Figure 1:**
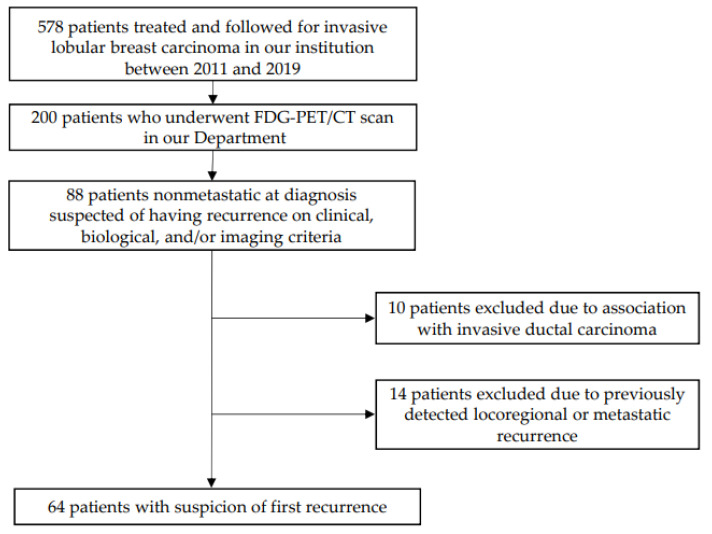
Flowchart of the study population. FDG-PET/CT: Positron Emission Tomography/Computed Tomography using 2-deoxy-2-[18F]fluoro-D-glucose.

A total of 64 patients were enrolled, as demonstrated by the flowchart in Figure 1. No recent previous PET/CT scan was performed in these patients. The characteristics of the study population are presented in Table 1. ILC were primarily grade II (75%), expressing estrogen receptors (95%), and HER2-negative (95%). The characteristics of the initial primary tumors and the values of tumor markers (Carbohydrate Antigen 15.3; CarcinoEmbryonic Antigen) prior to PET/CT scanning are presented in Table 2. No patient had metastasis at the time of the initial diagnosis. Age at initial ILC diagnosis was 60.3 ± 12.4 years (median 59.8, range 32–86 years). The average time from initial diagnosis of the primary tumor to suspicion of recurrence was 5.2 ± 4.1 years (median 4.3, range 1–18.8 years).

Recurrence was suspected for three main reasons (patients could have more than one of the following): (i) clinical symptoms (48%) such as onset of pain, a palpable lesion on examination, or alteration of overall health status; (ii) abnormal findings on conventional imaging (42%), requested during usual follow-up; or (iii) biological findings (30%), with elevated tumor markers. Among the 64 patients, 48 (75%) were diagnosed with recurrence by the oncologist: 7 had isolated local recurrence, 32 had metastatic recurrence only, and 9 had both local and metastatic recurrence. Metastatic recurrence affected mainly the bones (24/41; 58%), lymph nodes (14/41; 34%), and liver (10/41; 25%).

For 40 of the 64 patients (62.5%), a biopsy of the clinically palpable mass or of the lesion considered to be a likely site of recurrence on imaging was performed. In the remaining 24 patients (37.5%), there was either no target lesion, or no histopathological confirmation request when multiple metastases were observed on imaging exams other than PET/CT scans, in association with elevated tumor markers. In only five patients, the diagnosis of recurrence was based on PET/CT scan findings alone, without any other imaging or histopathological confirmation.

### 1.3. PET/CT Acquisitions

PET/CT scans were performed on one of two PET/CT machines in our center: Gemini TF, Philips Healthcare, Cleveland, Ohio, USA, or Discovery MI, GE Healthcare, Milwaukee WI, USA. After fasting for a minimum of 6 h, and when capillary blood glucose was <11 mmol/L, patients received an intravenous injection of 3 MBq/kg (0.08 mCi/kg) of F-18-FDG. After injection, patients rested quietly with a blanket and no stimuli. Whole-body scans were acquired in the supine position, from the vertex to the midthigh area, 60 ± 10 min after FDG injection, in 3D mode, with approximately seven bed positions of 1.5–2 min each (length: 18 cm with the Philips machine, 20 cm with the GE machine). Low-dose CT images without iodinated contrast injection were acquired with the following parameters: 160 mAs (Philips) or 60–300 mAs (GE), 120 kV, slice thickness 2.5 mm (GE) or 5 mm (Philips), 0.5 s per rotation, table speed 55 mm/rotation, pitch 0.7 (Philips) or 1.3 (GE). The low-dose CT acquisition was used to correct attenuation, for fusion and as a diagnostic aid. Immediately after CT, PET images were acquired in the caudocranial direction. CT data were resized from a 512 × 512 matrix to a 128 × 128 matrix for combination with PET data and image fusion.

### 1.4. PET/CT Analysis

PET/CT images were visually interpreted by five experienced nuclear medicine physicians with at least more than five years of experience in PET/CT imaging; they were aware of clinical setting, as well as data provided by the oncologists. They were analyzed using customary visualization protocols on dedicated workstations (Extended Brilliance Workspace 3.5, using Syntegra software (Philips), or AW Server software (GE)) with triangulation tools for 3D analysis. PET scans were read as positive when FDG uptake in a site was increased and higher than the surrounding background.

To calculate the average SUVmax of lesions related to ILC recurrence, the SUVmax value of the lesion with the highest intensity was retained for analysis.

### 1.5. Statistical Analysis

Quantitative variables are described as mean ± standard deviation if normally distributed, and as median with range if nonnormally distributed. Qualitative variables are described in numbers (percentages). Percentages are calculated excluding missing data. Comparisons between groups were performed using the Student’s t-test, Wilcoxon, chi-square, or Fisher’s exact tests, as appropriate. Median follow-up was determined by the inverse Kaplan-Meier method. Specific survival is defined as death due to cancer. Specific survival and median specific survival were calculated by the Kaplan-Meier method and compared between groups using the log-rank test. Specific survival rates were determined with their 95% confidence intervals (CI). Variables associated with recurrence were determined by univariate logistic regression. All tests were two-sided and *p*-value <0.05 was considered statistically significant. All analyses were performed using SAS version 9.4 (SAS Institute Inc., Cary, NC, USA).

## 2. Results

### 2.1. PET/CT Scan Performance

In total, among the 64 patients, 42 PET/CT scans were true positive, 14 true negative, 6 false negative, and 2 false positive. Sensitivity, specificity, positive predictive value, and negative predictive value of PET/CT for detection of a first recurrence of ILC in patients with suspected recurrence were, respectively, 87%, 87%, 95%, and 70%. The mean SUVmax value for the ILC recurrences was 6.4 ± 2.9 (median 5.8 [1.9–17.3]).

Among the 14 true negative scans, 10 showed no hypermetabolic activity, and 4 were classed as negative either because the findings were not suspect, e.g., mediastinal hilar lymph nodes or benign lung nodules, or because the findings were related to another pathology (e.g., a hypermetabolic lesion of the stomach or multiple hypermetabolic lymph nodes suggestive of lymphoma). Two pleural and one meningeal ILC metastases without increased FDG uptake on the PET/CT scan were found in three patients. However, the results of the PET/CT scans were true positive for these three patients, who also had hypermetabolic lymph node, bone, and liver recurrence. In several patients considered to have recurrence on the PET/CT scan, certain CT findings (mainly involving the bones) did not show any metabolic activity, but were associated with other hypermetabolic bone lesions that prompted a diagnosis of recurrence. The six false negative scans were local (*n* = 2), peritoneal (*n* = 2), meningeal (*n* = 1), and bladder recurrence (*n* = 1), respectively. All these patients underwent biopsy that confirmed the presence of ILC metastases, except for one patient with peritoneal recurrence identified on an abdominal CT scan. The two false positives corresponded to a presacral nodule related to a schwannoma and an inguinal mass revealing a follicular lymphoma. Other cancers were detected by PET/CT in four patients (two lymphoma, one lung, and one gastric cancers) but were classified as true negative for recurrence of ILC.

**Figure 2:**
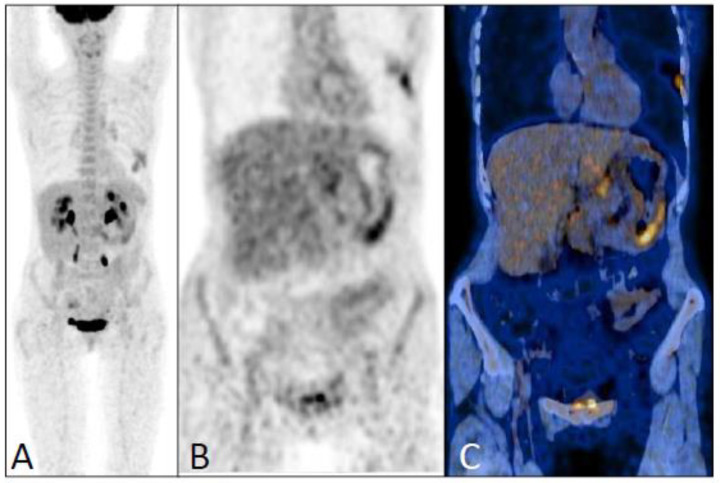
An FDG-PET/CT scan performed one year after the initial diagnosis of ILC (pT1bN0, SBR II, Ki67 = 30%) in a 56-year-old patient treated by aromatase inhibitor, due to a pyloric ulcer. Low increased FDG uptake in the thickened gastric wall was demonstrated on the maximum intensity projection (**A**), coronal PET (**B**), and PET/CT fusion (**C**) images. The increased uptake in the left lower chest was related to a pneumonitis. This patient died nine months later from disease progression of gastric adenocarcinoma.

Among the 40 patients with available histopathological confirmation of recurrence, there were 30 true positive PET/CT scans, five false negative, one false positive (schwannoma), and in four cases, other primary tumors of the lung (*n* = 1), the stomach (*n* = 1), (Figure 2) or lymphoma (*n* = 2) were discovered. The 30 true positive results were lymph node (*n* = 8), bone (*n* = 7), liver (*n* = 5), local (*n* = 4), cutaneous (*n* = 4), and pleural (*n* = 2) recurrences. The five false negative results corresponded to local (*n* = 2), meningeal (*n* = 1), peritoneal (*n* = 1), and bladder recurrence (*n* = 1).

### 2.2. Site of Recurrence on PET/CT Scans

**Figure 3:**
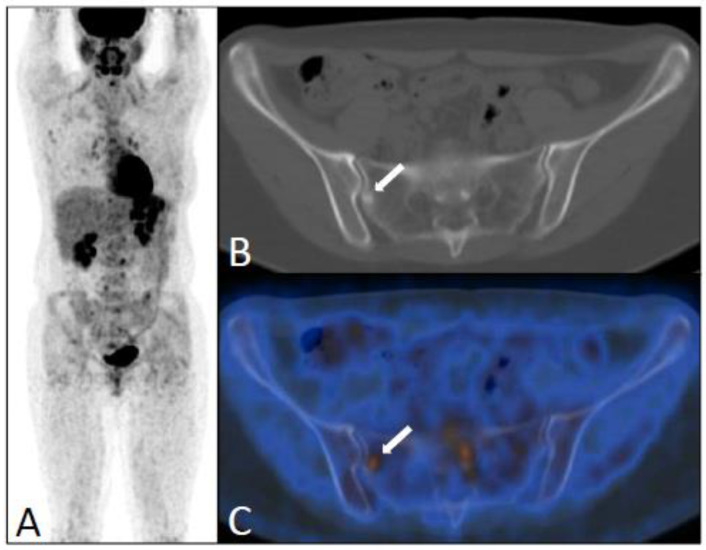
An FDG-PET/CT scan performed five years after the initial diagnosis of ILC (pT3N2, SBR II) of the right breast in a 37-y-old patient because of elevated tumor marker (CA 15.3 = 56) and pain in the thoracic spine. Maximum intensity projection (**A**) showing locoregional recurrence and bone metastases with low uptake. Axial CT (**B**) and PET/CT fusion images (**C**) of the pelvis showing a sclerotic lesion with low increased FDG uptake (white arrows). Hormone therapy was introduced (tamoxifen). This patient died five years later from disease progression with carcinomatous meningitis and uterine metastases.

**Figure 4:**
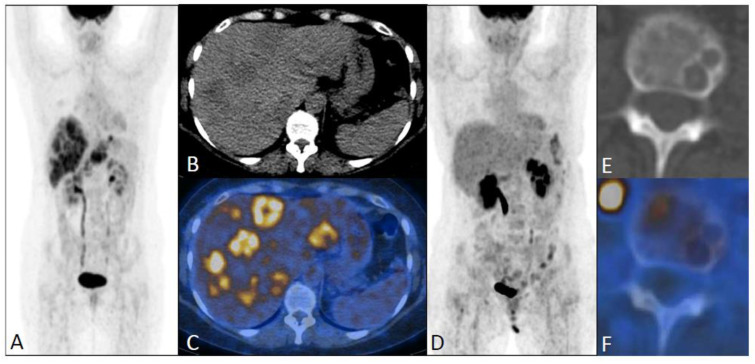
An FDG-PET/CT scan performed three years after the initial diagnosis of ILC (pT2N1, SBR II) in a 52-year-old patient treated by aromatase inhibitor, due to elevated tumor marker (CA 15.3 = 248). Maximum intensity projection (**A**), Axial CT (**B**), and PET/CT fusion (**C**) images showing metastatic spread to the liver, confirmed by biopsy. Maximum intensity projection (**D**) showed metabolic response after three cycles of chemotherapy. However, on this follow-up PET/CT scan, lytic bone metastases with no FDG-avidity were found, as shown on axial CT (**E**) and PET/CT fusion (**F**) images. This patient died 18 months after the diagnosis of recurrence.

**Figure 5:**
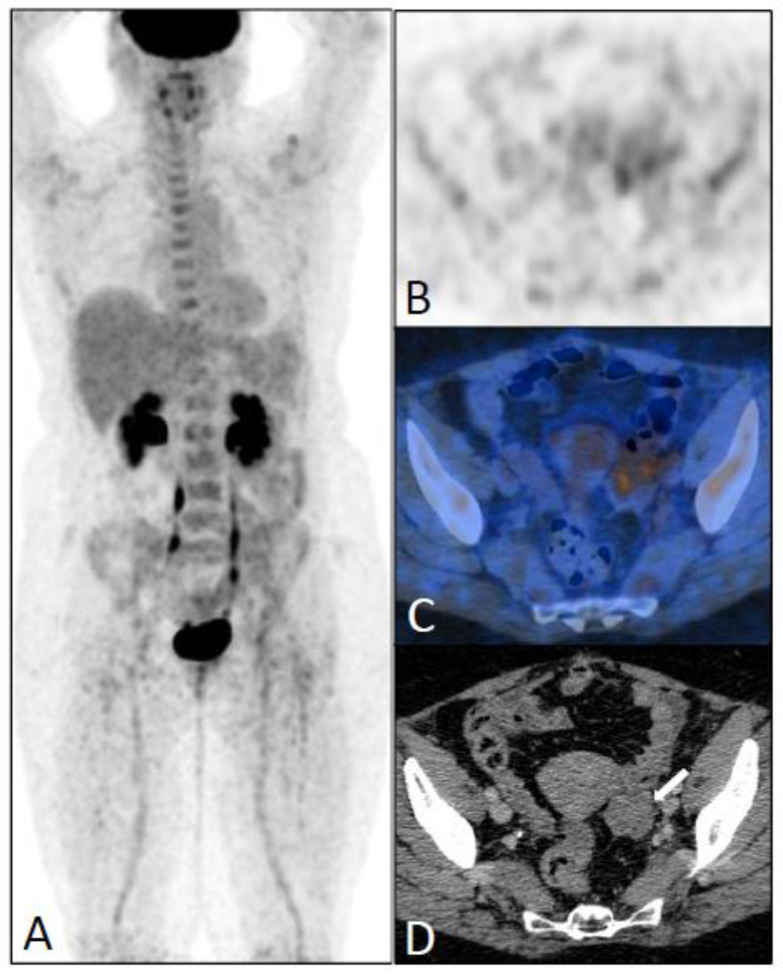
An FDG-PET/CT scan performed eight years after the initial diagnosis of ILC (pT3N1, SBR II) in a 55-year-old patient, due to elevated tumor marker (CA 15.3 = 249). Maximum intensity projection (**A**), Axial PET (**B**), and PET/CT fusion (**C**) images showing a faintly increased FDG uptake not described in the report. A left uterine mass (white arrow) associated with a peritoneal effusion was found on a CT scan (**D**) performed one week after the PET/CT scan. Peritoneal and ovarian metastases were confirmed by laparoscopy and chemotherapy was introduced. This patient died from disease progression in the peritoneum nine years after the diagnosis of recurrence.

Distant recurrences were observed in bone (*n* = 24, 50%) (Figure 3), extraaxillary ipsilateral lymph node (*n* = 14, 29%), liver (*n* = 10, 20%) (Figure 4), lung and pleura (*n* = 2), meninge (*n* = 2), and peritoneum (*n* = 1) (Figure 5). Bone lesions with hypermetabolic activity were mainly sclerotic (*n* = 8), mixed (*n* = 6), or lytic (*n* = 6), or were not clearly visible on CT scans or “occult” (*n* = 4).

### 2.3. Relation between PET/CT Findings, Tumor Stage, and Histological Prognostic Factors

The rate of recurrence was higher in patients who initially had stage pT3 or pT4 lesions compared to those with initial-stage pT1 or pT2 (94.4% vs. 66%) (Table 2).

No association between SUVmax and histological prognostic factors (Ki67, mitotic index, or grade of mitosis, included in the score of Scarff Bloom Richardson (SBR)) was found (Table 2).

We did not investigate the association between SUVmax and expression of hormone receptors and/or HER2 expression, given the high proportion of ILC expressing estrogen receptors and HER2 negativity in the study population.

### 2.4. Modifications of Treatment following the PET/CT Findings

In total, 44/48 patients (92%) had a change in the treatment following the PET/CT findings. The four cases of recurrence that did not lead to a change in treatment were due to either patient’s refusal, or to a major impaired general condition. In 41 patients with metastatic recurrence, chemotherapy regimens were generally introduced (*n* = 29), potentially combined with hormone therapy, targeted therapy, or immune therapy. Surgery and/or radiotherapy were administered in eight patients and hormone therapy alone in four patients. The three remaining patients belonged to the group of patients with only local recurrence. Only one patient with local recurrence had a slight modification of treatment, consisting in a different hormone therapy.

### 2.5. Association between PET/CT Findings and Survival

**Figure 6:**
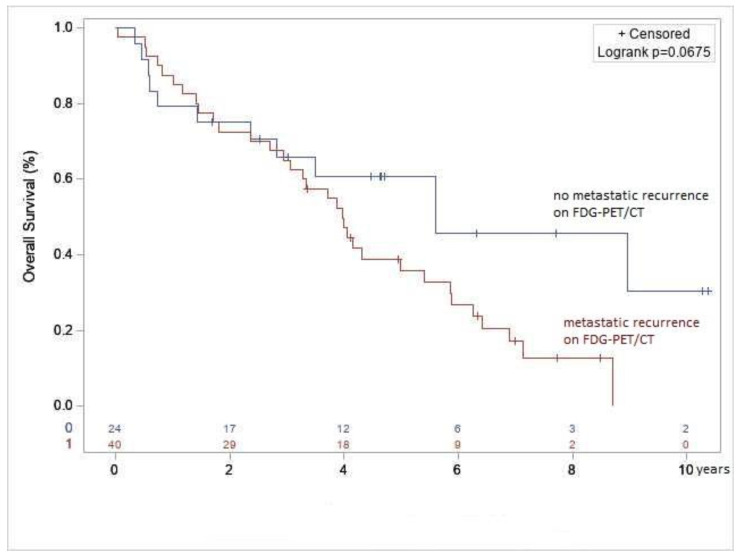
Kaplan-Meier analysis for specific survival in patients with metastatic recurrence and patients with no metastatic recurrence or locoregional recurrence on PET/CT imaging.

Median specific survival of patients with a metastatic recurrence on PET/CT scan was 4.0 years, 95% CI [2.9–5.4], whereas median specific survival was not reached for those with no or local recurrence on PET/CT (*p* = 0.067). Five-year survival was 35.9%, 95% CI [21.1%−50.8%] for those with metastatic recurrence on PET/CT, vs. 60.8%, 95% CI [37.9–77.5] for those with no or isolated local recurrence (Figure 6). On the cox univariate analysis, hazard ratio was significant in patients with metastatic recurrence on PET/CT (1.9 [0.95–3.75]; *p* = 0.072). Among the seven patients with local recurrence, four are still alive, but one patient who was still alive more than five years after the recurrence developed peritoneal and bone metastases three years after the first local recurrence. One patient had repeated local recurrences without distant progression. Three patients died, but these deaths were not related to the ILC. Among the 16 patients diagnosed not to have recurrence, four died, of whom only one from meningeal metastasis of the ILC; this death occurred 5.4 years after the first suspicion of recurrence. In two patients, cause of death was pleural progression of gastric adenocarcinoma and meningeal progression of bronchopulmonary adenocarcinoma.

## 3. Discussion

The present study evaluates specifically the diagnostic performance of PET/CT scanning in cases of suspected recurrence, in a population of 64 consecutive breast cancer patients treated 5.2 ± 4.1 years earlier for ILC. Follow-up was consistent, and performed in a single institution, from the time of initial diagnosis to first suspicion of recurrence. We show that PET/CT scan performs well (sensitivity and specificity values of 87%) for the detection of ILC recurrence. SUVmax at the sites of ILC recurrence was generally high, at an average of 6.4 ± 2.9. This confirms other reports notably with a signal-to-noise ratio sufficiently high to enable detection of recurrent lesions, even though it has been shown that the SUVmax of primary tumors of ILC is significantly lower than that of IDC (Table 3) [8,9,10,12,13,14,15]. To calculate the average SUVmax of lesions related to ILC recurrence, the SUVmax of the lesion with the highest intensity was retained for analysis. The lower FDG-avidity of ILC could explain the lower sensitivity of FDG-PET/CT scan as compared to IDC [18], especially for small lesions. This point is especially true for lesions localized in the abdomen with greater background noise than in the thorax, for example, such as peritoneal or ovarian lesions, more frequently observed with ILC than with IDC. In addition to the usual secondary sites associated with breast carcinoma, an infiltrating metastatic pattern has been described, towards the gastrointestinal tract, the ovaries, and towards the peritoneal and retroperitoneal spaces and leptomeninges [5,6,20,21,22,23,24]. These metastatic sites generally become clinically apparent at a late stage, and are known to be difficult to identify on PET/CT scan (Figure 5). The metastatic sites falsely negative on PET/CT scans observed in the present study, namely pleural, meningeal, and peritoneal lesions, are in line with previous literature reports [5,6,20,21,22,23,24]. Furthermore, it is noteworthy that no hypermetabolic findings of the pleura were observed in the two patients who had positive pleural puncture, which could be explained by diffuse pleural disease without any focal lesion. In eight patients, two local lesions and six distant lesions with no hypermetabolic activity were found to have a lobular histology according to the biopsies. Among these eight patients, three had lobular histology on pleural and/or lumbar puncture without associated metabolic activity, but their PET/CT scans were true positive due to the presence of other distant hypermetabolic findings suggestive of recurrence. Of the five patients with false negative scans, three were receiving hormone therapy at the time of PET/CT, which may have reduced metabolism in tumor cells, as previously described by Champion et al. [25]. These authors reported that after withdrawal of endocrine therapy in nine asymptomatic patients with initially negative PET/CT scans, the results switched to positive on a second PET/CT scan performed a few weeks later, enabling the detection of breast cancer recurrence. It should be noted that ILC expresses predominantly hormone receptors (95% in the present study), which generally leads to administration of adjuvant hormone therapy.

It has previously been shown that bone lesions are less FDG-avid in ILC than in IDC, and more frequently sclerotic [26,27] (Figure 3). In the present study, observed bone lesions were predominantly sclerotic on CT, confirming the findings of Dashevsky et al. [26], with a distribution of sclerotic, mixed, or lytic lesions or with CT occult bone metastases similar to that reported by Orevi et al. [19] (Figure 4).

The stage of the initial ILC is probably worth taking into consideration when there is a suspicion of recurrence, because in the present study, although patients with initial stage three or four accounted for only 28% of the total number of patients, 94% of them were deemed to have recurrence, compared to only 66% of the patients with initial stage one or two ILC, as previously reported by Park et al. [28] It has been reported that elevated tumor markers should prompt complementary investigations, and PET/CT performs well in ILC [25]. Accordingly, in the present study (Table 2), 26 patients out of 29 were diagnosed with recurrence by the oncologist when CA 15.3 was greater than 35 kU/L prior to PET/CT (*p* = 0.03). Among these 26 patients with CA 15.3 values above normal and progressive disease, 23 had PET/CT findings in favor of progression of ILC, and the three other patients had false negative PET/CT results, for local (*n* = 1), peritoneal (*n* = 1), and bladder (*n* = 1) recurrences.

Median specific survival was around four years in patients diagnosed with metastatic recurrence on PET/CT imaging and was not reached in patients with no metastatic recurrence. Among the patients with isolated local recurrence, only one patient progressed to metastatic disease distant from the local recurrence (around three years later). Given the low number of patients with local recurrence, and their good prognosis, we compared PET/CT performance between the group of patients with metastatic recurrence and the group with no or only local recurrence. The statistical comparison showed that PET/CT had a good prognostic value in patients with no metastasis (*p* = 0.067). This is in line with the findings of Cochet et al. in 63 patients with suspected recurrent breast cancer, regardless of histological type [29].

We did not observe any relation between SUVmax and features reflecting tumor proliferation, such as Ki67 (*p* = 0.23) or mitotic index (*p* = 0.66), but this could be explained by the small sample size. Indeed, Ki67 and mitotic index were available in only 22 and 38 patients respectively. Therefore, we were unable to replicate the findings of Choi et al. [30], who reported a significant relation between SUVmax of ILC and Ki67 in 81 patients, with a higher mean SUVmax observed when Ki67 was higher than 15% (*p* = 0.07).

The present study suffers from some limitations. First, we were unable to establish a significant relationship between PET/CT results and the histological prognostic factors of the initial primary tumor due to the retrospective nature of the study. Indeed, missing data mainly concerned patients treated a long time ago for the primary tumor (the time interval between initial diagnosis of ILC and the first suspected recurrence reached up to 18.8 years). The final diagnosis of recurrence was established by the oncologist, taking into account the results of the PET/CT scan, and therefore, there is potential for methodological bias due to the absence of a real “gold standard” diagnostic method for recurrence. Moreover, this observation might suggest an overestimation of change in treatment considering that recurrence was not only detected by PET/CT, especially in the subgroup of patients with abnormal findings on conventional imaging. However, whole-body PET/CT generally offers the opportunity to restage disease and allows a better characterization of these findings. In the present study, PET/CT was the only imaging exam performed to detect recurrence in five patients (more than 10 bone hypermetabolic lesions observed fitted with bone morphologic lesions on the CT component of the PET/CT scan in four of them). Only one out of these five patients was symptomatic. Furthermore, histopathological confirmation was not obtained for all patients presenting hypermetabolic lesions suggestive of metastatic recurrence, but might have made it possible to confirm the lobular subtype of these metastatic lesions (biopsy was not performed systematically). It is also noteworthy that the indication for the biopsies that were performed was likely guided by the results of PET/CT. However, histopathological confirmation of the site of suspected recurrence was available in 40/64 patients (62.5%). Among these patients, ILC recurrence was confirmed in 30 patients, and PET/CT also enabled detection of other cancers in four cases (lymphoma, lung, and gastric cancers).

A prospective study comparing the findings of the initial PET/CT and the PET/CT scan performed at the time of first suspected recurrence of ILC would be of interest, but this comparison could not be performed in our present study due to the small number of patients for whom both scans were available (*n* = 6). A study of PET/CT with F-18-Fluciclovine would also be of interest, since it has been reported that breast cancers have a higher level of avidity for F-18-Fluciclovine than for FDG [31,32]. Fluorofuranyl norprogesterone (F-18-FFNP), which has an attractive signal-to-noise ratio in the initial staging of ILC, could be of use in the detection of ILC recurrence [33]. Although no study to date has evaluated the utility of FES (16α-[F]fluoro-17β-estradiol) for staging of ILC, the high rate of the expression of estrogen receptors in ILC has prompted evaluations of this tracer able to detect more metastatic lesions of ILC than FDG [34,35].

## 4. Conclusions

The present study demonstrates that FDG-PET/CT scanning is a valuable and reliable tool for the detection of recurrence of ILC, although certain specific characteristics of ILC can impair diagnostic performance, notably peritoneal or meningeal metastatic lesions, which are difficult to detect on PET/CT, and/or lesions with low FDG-avidity. The absence of metastatic lesions on PET/CT scan appears to be associated with a better prognosis in terms of specific survival.

## Figures and Tables

**Table 1:** Characteristics of the study population.

Initial treatment		
Surgery: 61 (95.3%)	Radiotherapy: 52 (81.3%)	
Chemotherapy: 45 (70.3%)	Hormone therapy: 55 (85.9%)	
pTNM		
pT1: 21 (32.8%)	pN0: 21 (32.8%)	
pT2: 23 (36.0%)	pN+: 41 (64.2%)	
pT3: 15 (23.4%)	M0: 64 (100%)	
pT4: 3 (4.7%)		
NA: 2 (3.1%)		
SBR grade	Mitoses grade	
I: 8 (12.5%)	1: 47 (73.5%)	
II: 48 (75%)	2: 7 (10.9%)	
III: 6 (9.4%)	3: 3 (4.7%)	
NA: 2 (3.1%)	NA: 7 (10.9%)	
Mitotic indices mean: 2.7 [0.1–13.2]	NA: 25 (39.1%)	
Ki67 mean: 24% [5–60]	NA: 42 (65.6%)	
Hormonal receptors		
ER positive: 61 (95.3%)	NA: 1 (1.6%)	
PR positive: 47 (73.5%)	NA: 3 (4.7%)	
HER2 positive: 3 (4.7%)	NA: 1 (1.6%)	
Criteria for suspicion of recurrence (more than one criterion possible for each patient)		
Clinical: 31 (48.4%)		
Imaging: 27 (42.2%)		
Tumor markers: 19 (29.7%)		
Tumor markers elevated	Value	
CA 15.3: 29 (45.3%)	mean: 160.1 kU/L (N ≤ 35.0)	NA: 9
CEA: 22 (34.4%)	mean: 23.5 μg/L (N ≤ 5.0)	NA: 14

SBR: Scarff-Bloom-Richardson; ER: estrogen receptors; PR: progesterone receptors; HER2: human epidermal growth factor receptor 2; CA 15.3: carbohydrate antigen 15.3; CEA: carcinoembryonic antigen.

**Table 2:** Comparison of prognostic powers of various histopathological and biological parameters for diagnosing first recurrence in 64 patients with suspected first ILC recurrence.

	No-Recurrence N = 16	Recurrence N = 48	*p*-Value
pT			
T1	9	12	0.07 ^a^
T2	6	17	
T3	1	14	
T4	0	3	
Not available	2		
pN			
N0	6	15	0.97 ^a^
N1	5	17	
N2	4	11	
N3	1	3	
Not available	2		
Mitotic index			
≤3	10	17	0.27 ^a^
>3	2	10	
Not available	4	21	
Mitoses			
Grade 1	12	35	1 ^a^
Grade 2	1	6	
Grade 3	0	3	
Not available	3	4	
Ki67			
≤20%	7	5	0.23 ^a^
>20%	3	7	
Not available	6	36	
Tumor markers			
CA 15.3 ≤ 35 kU/L	9	17	0.03 ^b^
CA 15.3 > 35	3	26	
Not available	4	5	
CEA ≤ 5 μg/L	8	21	0.16 ^a^
CEA > 5	2	19	
Not available	6	8	

^a^ Fisher’s exact test; ^b^ chi-squared test.

**Table 3:** Comparison of SUVmax in ILC and IDC available in the literature (med: median).

Authors	Number of ILC/Number of Patients	SUVmax ILC	SUVmax IDC	*p*-Value
Crippa [8], 1998	18/86	3.8 (med)	5.6	0.004
Avril [9], 2001	14/50	2.2 ± 1.4	3.7 ± 2.2	0.003
Ueda [10], 2008	32/145	1.4 ± 1.5	4.8 ± 3.5	0.004
Groheux [12], 2011	15/132	3.4 (med)	6.6	0.0001
Koolen [13], 2012	23/214	4.6 (med)	7.1	0.001
Groheux [14], 2015	7/171	3.7 ± 1.2	7.4 ± 5.2	0.008
Jung [15], 2015	32/105	2.0 ± 1.7	3.9 ± 4.0	0.032

## Data Availability

The datasets generated and/or analysed during the current study are not publicly available due to the French law rules but are available from the corresponding author on reasonable request.

## Abbreviations

ILC	invasive lobular carcinoma
IDC	invasive ductal carcinoma
PET/CT	positron emission tomography/computed tomography
FDG	2-deoxy-2-[18F]fluoro-D-glucose
MRI	magnetic resonance imaging
SUVmax	Standard Uptake Value maximum
SBR	Scarff Bloom Richardson
95%CI	95% confidence interval
